# Supplementary material for: Sensorimotor computation underlying phototaxis in zebrafish
Source: Nat Commun. 2017 Sep 21;8:651. doi: 10.1038/s41467-017-00310-3 (PMC5608914; doi:10.1038/s41467-017-00310-3)
Supplement: Supplementary file 1 — Supplementary Information [file 41467_2017_310_MOESM1_ESM.pdf]

File Name: Supplementary Information

Description: Supplementary Figures, Supplementary Methods and Supplementary References.

File Name: Supplementary Movie 1

Description: Eye/tail coordination. Sequence of spontaneous ocular saccades and swim-bouts in a partially restrained larva, showing eye/tail coordination. The blue and red curves show the tail deflection signal and the gaze angle, respectively. The animal is submitted to whole-field illumination.

File Name: Supplementary Movie 2

Description: Brain-scale peri-saccadic neural activity. Images were obtained using one-photon light-sheet imaging (1h recording, 20 brain sections, one stack per second) with simultaneous monitoring of the eyes (bottom-left panel). Color encodes fluorescence transients of individual voxels: blue for rightward saccades, red for leftward saccades. The sequence was obtained by averaging over multiple saccades.

File Name: Supplementary Movie 3

Description: 3D functional maps of gaze-tuned neurons. These maps were computed through regression between the gaze angle traces and individual voxel flurescence activity. Colors encode the level of tuning (Z-score) to gaze position (left) and velocity (right). Seven fish were registered to produce these functional 3D maps.

File Name: Supplementary Movie 4

Description: Comparison of gaze-tuned, self-oscillatory, and visual response 3D maps. Each 3D map was obtained through regression-based functional identification followed by 3D registration on a reference brain, as described in Supplementary Methods. From left to right : gaze-tuned populations, self-oscillatory hindbrain population, visually responsive neurons. This video shows that the HBO is engaged in all three processes.

File Name: Peer Review File

Description:

Supplementary Methods

Zebrafish lines and maintenance

All experiments were performed on zebrafish nacre mutants, aged 5 to 7 days post-fertilization (dpf). For functional imaging, embryos were exposed to 1-phenyl-2-thiourea at 0.2 mM (PTU, Sigma-Aldrich), from 12 to 24 hpf, in order to further reduce skin pigmentation. Larvae were reared in Petri dishes in E3 solution on a 14/10h light/dark cycle at 28°C, and were fed powdered nursery food every day from 6 dpf. Calcium imaging experiments were carried out on larvae expressing the calcium indicator GCaMP6f under the control of the nearly pan-neuronal promoter *elavl3*. This Tg*(elavl3:GCaMP6f)a12200* line was generated by PCR amplification of the GCaMP6f open reading frame (from pGP-CMV-GCaMP6f, Addgene plasmid 40755[1]) with forward primer ataACTAGTgccaccATGGGTTCTCATCATCAT and reverse ataCCGCGGcTCACTTCGCTGTCATCATTTGTAC (restriction site and coding sequences in upper case, respectively). This fragment was cloned into a plasmid with Tol2 arms flanking an upstream attR1-R2 cassette and the insertion site using restriction enzymes SpeI and SacII. Previously described *elavl3* (*HuC*) *cis*-regulatory elements[2]. were placed upstream via LR recombination (Invitrogen) with an attL flanked *elavl3* entry clone. The resulting plasmid was then co-injected into 1-cell stage embryos at a concentration of 30 ng/µL with Tol2 transposase mRNA at a concentration of 30 ng/µL. A single founder was selected based on high and spatially broad expression. Outcrossing this founder generated ~50% GCaMP6f-positive embryos, which were selected to establish the line.

Optogenetic experiments were performed with double transgenic fish Tg(*elavl3*:GAL4-VP16)[3] X Tg(*UAS*:ChR2-H134R-mCherry)[4]. All experiments were approved by *Le Comité d'Éthique pour l'Expérimentation Animale Charles Darwin* (02601.01).

Behavioural assays

Zebrafish larvae were embedded in a drop of 2 % low melting point agarose (UltraPure LMP Agarose, Invitrogen) deposited on a petri dish. Agarose was removed from regions around the eyes for phototaxis assays, and from the eyes and tail for assessing saccades/swim turns coordination. The petri dish was then filled with E3 medium. We visually checked for robust saccadic dynamics using a dissection microscope before placing the larvae at the center of a 5 cm in diameter frosted glass disk lit from below with an infra-red LED and positioned inside a dark chamber at 28 °C. Eye and/or tail movements were recorded using an infrared sensitive Flea3 USB3 Camera (FL3-U3-13Y3M-C, Point Grey Research, Richmond, BC, Canada) with an adjustable macro lens (Zoom 7000, Navitar, USA) with pixel-encoded timestamps.

All visual assays reported in the manuscript were performed using stimuli in the blue domain in order to prevent the fluorescence signal from being polluted by visual stimuli during neuro-imaging experiments. Controlled stereo-visual stimulation was obtained using two LEDs (LED465E - Thorlabs) separated by a partition and projected onto the frosted screen, so that each LED delivered a uniform illumination to one side of the animal's visual field of view. Analog outputs of a DAQ device (USB6000 - National Instrument) were used to control the intensity of the visual stimulation in the range $0 < I < I_{max}$, with I_{max}=60 µW/cm^2. We used MATLAB (MathWorks) for image analysis and on-line control of the visual stimuli.

Saccades/swim-turns coordination experiments were performed under whole-field constant illumination. We recorded the eyes and tail kinetics over periods of ~2h at 200 frames/s. Image analysis was performed offline. We extracted the orientation of each eye by computing the angle between the equivalent-ellipse minor axis (i.e. the eye optical axis) and the medial-lateral axis of the fish (right: θ_r, left: θ_l), from which we computed the gaze angle defined as $\theta_{gaze} = (\theta_r - \theta_l)/2$. The mean post-saccadic gaze angles $< \theta_{gaze\ right} >$ and $< \theta_{gaze\ left} >$ were used to define a characteristic gaze range (Supplementary Fig. 1a) from which we computed the normalized gaze angle:

$$g = (2 * \theta_{gaze} - < \theta_{gaze\ right} > - < \theta_{gaze\ left} >)/(< \theta_{gaze\ left} > - < \theta_{gaze\ right} >) \quad (1)$$

The onset of each saccade was further extracted with a time resolution of ~5ms based on the time-derivative of the gaze signal. Reorienting saccades were defined such that $g * \Delta g < 0$ where Δg is the saccade induced gaze shift and g is the gaze value prior to the saccade. Secondary saccades are in turn defined such as $g * \Delta g > 0$.

To evaluate swim bout orientation, we binarized the image of the tail region (from the caudal border of the swim bladder to the tip of the tail) and computed at each time the moment of area with respect to the animal rostro-caudal axis, *i.e.* the mean distance to the axis computed over the tail pixels. To allow for across-samples comparison, this quantity was normalized by the tail length yielding an adimensional parameter *m(t)* characterizing the instantaneous lateral excursion of the tail. The time derivative of this signal was used to identify and separate discrete tail bouts (Supplementary Fig. 1c). We computed for each event the onset and offset times τ_{on} and τ_{off} as well as a turning score defined as $M = \int_{\tau_{on}}^{\tau_{off}} m(t).dt$. Large positive (negative) M values corresponded to strong leftward (rightward) attempted turns. The normalized gaze angle associated with a given tail-bout was defined as its value evaluated in the middle of the bout, $g\big((\tau_{on} + \tau_{off})/2\big)$.

To assess stereo-visually driven gaze bias, the fish were first submitted to a period of 10 minutes of habituation (whole-field illumination at maximum intensity) followed by 100 cycles comprising, (1) 1 min of whole-field illumination, (2) 25 s of extinction of one (either left or right) LED, (3) 5 s during which the LED intensity was linearly increased up to the its initial value. This sequence was repeated 50 times for each eye, either alternately or sequentially. The gaze orientation signal was extracted offline. To evaluate light-induced bias in gaze orientation, we computed the trial-averaged gaze shift towards the illuminated eye during periods of unilateral stimulation. We thus calculated, for each fish :

$$\Delta\theta = < \int_{t_{on}}^{t_{on}+30s} (g(t) - g(t_{on}))\,.\,dt >_{right\ extinction} - < \int_{t_{on}}^{t_{on}+30s} \big(g(t) - g(t_{on})\big)\,.\,dt >_{left\ extinction}$$

where the first (resp. second) term is averaged over the 50 left (resp. right) extinction periods, t_{on} being the onset times of the extinction. To assess the significance of the light-induced bias for each fish, we calculated the same quantity for 100 randomly distributed times t_{on}. This procedure was replicated 1000 times, yielding a distribution of $\Delta\theta_{null}$ centered on zero (standard deviation σ) that characterized the expected probability distribution under the null hypothesis. A two-tailed test with α = 0.05 was applied to assess visual-induced gaze bias: $\Delta\theta > 2 * \sigma$ (resp. $\Delta\theta < 2 * \sigma$) was interpreted as a positive (resp. negative) phototactic behaviour (Supplementary Fig. 2g).

To check that the light-induced gaze bias resulted in a consistent swim bias, we ran a series of experiments using the same stimulation protocols on fish whose both eyes and tail were freed. Both the gaze and tail-defection signals were extracted offline. We then computed the trial-averaged swim bias during periods of unilateral stimulation by calculating, for each fish :

$$\Delta m = < \int_{t_{on}}^{t_{on}+30s} m(t)\,.\,dt >_{right\ extinction} - < \int_{t_{on}}^{t_{on}+30s} m(t).\,dt >_{left\ extinction}$$

The significance of the light-induced swim bias for each fish was quantified as described above for the gaze-bias ($\Delta m > 2 * \sigma$). With this criterium, the fish that exhibited a light-induced gaze bias towards the light source, systematically showed a significant swim-bias towards the light (N=5).

Virtual-reality assays of spatio-temporal gaze bias were performed as follows. Larvae were first subjected to a minimum of 30 min of visual habituation at maximum illumination intensity. A 20 min-long recording was acquired during this initial period. The 5th and 95th percentile of the gaze distribution during this neutral run - noted $< \theta_{gaze\ right} >$ and $< \theta_{gaze\ left} >$, respectively - characterized the typical gaze range, from which we computed the normalized gaze angle *g(t)* as in equation (1) for all subsequent runs. The whole-field illumination intensity was then modulated in real-time according to the extracted gaze angle with a refresh rate of 100 Hz. The intensity delivered to the fish was a sigmoid function of the normalized gaze: $I(g) = \alpha + 0.5\,\beta\,(1 +$

$erf(2g)$), where erf is the error function. Notice that the gaze signal *g(t)* was dynamically smoothed with a non-linear filter to eliminate fluctuations due to image analysis, which would otherwise result in flickering visual stimulation, while preserving the rapid transitions associated with saccades. We successively imposed a rightward-biased ($\alpha = 0, \beta = I_{max}$) and a leftward-biased ($\alpha = I_{max}, \beta = -1$) intensity profile. We extracted from these runs the time fraction f_{light} during which the gaze was oriented towards the light ($I > I_{max}/2$). To assess the significance of the light-induced bias, we implemented a bootstrap approach. The neutral run (constant illumination) was randomly split into 100 segments, which were distributed into two packets. The segments were 10 s-long in average, *i.e.* of the order of the gaze fixation duration such that they could be considered as statistically independent. We computed the mean of the time-fraction for which $g > 0$ for the first packet, and $g < 0$ for the second packet. We replicated this procedure for 1000 different segmentations of the neutral signal. The resulting distribution of the time-fractions was gaussian-like (standard deviation σ) and centered on 0.5; it reflected the statistical distribution of biases expected under the null hypothesis for a 1000 s long run. For an experiment of total duration T_{bias}, the distribution was thus expected to have a standard deviation of $\sigma * \sqrt{1000/T_{bias}}$. We used this latter value to perform a two-tailed test with α = 0.05 (critical z-value of 2) on the light-biased data to assess the significance of visually induced gaze bias (Supplementary Fig. 2i-k).

Light-sheet functional imaging

Volumetric functional recordings were carried out using custom-made one-photon and two-photon light-sheet microscopes whose optical characteristics have been detailed elsewhere[5]. Larvae were mounted in a 1 mm diameter cylinder of low melting point agarose at 2 % concentration. The eyes region was systematically excluded from the scanned volume. One-photon illumination was used for the eyes-free experiments as they require significant post-processing to correct saccade-induced tissue deformation that proved difficult to implement in two-photon recordings. However, four recordings under 2P light-sheet imaging were performed to

check the absence of any spurious effects associated with the background light level under 1P imaging (Supplementary Fig. 5a). Acquisition rate was set at 1 stack per second (20 brain sections, 6 µm inter-slice separation), and typical recordings lasted for ~1 h. In the eyes-fixed experiments (identification of the self-oscillatory circuit and characterization of the visual response), the animal was fully embedded in the agarose gel, which entirely suppressed the saccadic dynamics. In this configuration, the animal was weakly paralyzed by adding Pancuronium bromide at 0.3 mg/mL in the agarose solution in order to further minimize movement artifacts. These eyes-fixed experiments were performed in the two-photon regime to mitigate perturbation of the visual system by the laser scanning[5]. Recordings were performed at 1 stack per second (in general 10 brain sections, 9 µm inter-slice separation) and lasted for 1 to 2h.

Image pre-processing, soma and neuropil regions segmentation as well as calcium transient ($\Delta F/F$) extraction were performed offline using MATLAB, according to the workflow previously reported[5,6]. A non-rigid 2D registration was further implemented in eyes-free recording to correct saccade-induced tissue deformations using the Matlab built-in functions *registration* and *imwarp.*

During neuro-imaging, eyes movements were recorded from below using an infrared sensitive Flea3 USB3 Camera (FL3-U3-13Y3M-C, Point Grey Research, Richmond, BC, Canada) with an adjustable macro lens (Zoom 7000, Navitar, USA) with pixel-encoded timestamps. The setup was lit from below with an infra-red LED at 850 nm. Image acquisition, mirror scanning, objective motion and eyes movements monitoring were synchronized using a D/A multifunction card (NI USB-6259 NCS, National Instruments) and a custom-written program in LabVIEW (National Instruments). Both the behaviour and neuroimaging cameras were operated at 20 Hz.

In order to map gaze-tuned neuronal populations, we performed a regression analysis on eyes angular position and velocity. The regression was performed voxel by voxel, after binning the images down to a pixel size of 0.8x0.8 μm^2. We manually selected the brain region, excluding the

areas rostral to the cerebellum in which the saccade-induced tissue deformation precluded consistent signal extraction. The method used to compute the Z-score of every voxel essentially followed the approach proposed by Miri *et al.*[7] We used for the eye velocity regressor a vector consisting of +(-)1 at the onset times of a leftward (rightward) saccades, and zeros elsewhere (for saccade onset detection, see paragraph *Saccades/swim-turns coordination*). This discrete signal offered a noise-free proxy for the time-derivative of the eye angular position.

The mean peri-saccadic response of the key functional regions shown in Figure 2 and Supplementary Fig. 3 were calculated over a 10 s-long window. Averaging was performed over ~150 saccades, excluding time-segments in which movements or another saccade occurred during the 10 s window. Before averaging, fluorescence signals from individual voxels were over-sampled to match the eye-tracking frame-rate (20 Hz). To prevent mixing pre- and post-saccadic activities, this interpolation was performed independently on the pre- and post-saccadic phases.

The neuronal assembly engaged in the self-generated oscillations in eyes-fixed experiments was delineated based on activity correlation analysis (Supplementary Fig. 4). We first performed a morphological segmentation that yielded a DF/F signal for all the neurons in the recorded region[5,6]. We then calculated the Pearson correlation matrix, corrected by the uniform correlation noise[5], of all pairs of neurons within a 100x60x27 μm^3 volume positioned relative to morphological landmarks such as to encompass the 4 most posterior clusters of the rostral hindbrain gaze-tuned population (see Supplementary Fig. 4a). We then selected the 10 pairs of neurons with the largest anti-correlation. These neurons were separated into two groups, based on their position relative to the medial axis. The mean $\Delta F/F$ signals of the left and right clusters provided us with two reference signals (Supplementary Fig. 4d). We then performed a regression analysis on a voxel basis, as described above, using these reference signals as regressors to produce the self-oscillatory functional map shown in Figure 3b-c.

The left and right HBO signals shown in Figures 3-5 were computed as the mean signal of individual HBO neurons selected on the basis of their correlation with the left-minus-right reference signal (Pearson correlation coefficient threshold +0.2 and -0.2). This selection method yielded a total of 348.4 ± 16.1 neurons (std 142.5, N=78 fish).

We used blue LEDs (LED465E - Thorlabs, 5 cd, 15° aperture angle) for visual stimulation. The stimulus intensity was calibrated by measuring the radiance at a similar distance as a function of the applied voltage. For asymmetrical visual stimulation, two LEDs were positioned symmetrically outside of the chamber at 45° and 4.5 cm from the fish eyes. Each LED delivered a visual intensity of 20 $\mu W/cm^2$. The flash stimulation was performed using a single LED positioned inside the chamber 4.5 cm in front of the fish eyes[5] and delivering 31 $\mu W/cm^2$, while symmetric alternating stimulation was obtained by projecting one LED, delivering 8 $\mu W/cm^2$, onto a 2x2 cm^2 diffusive screen positioned 0.55 cm below the fish. The blue photons were filtered out from the imaging system using a bandpass filter (525/39 nm Brightline, Semrock). Image acquisition and visual stimulation were synchronized using a D/A multifunction card (NI USB-6259 NCS, National Instruments) and a custom-written program in LabVIEW (National Instruments).

The unilateral visual response map (Figure 4 and Supplementary Fig. 5b) was obtained by delivering alternating asymmetric ON-OFF stimuli. The intensity time-sequence of the stimulus delivered to each eye were used as regressors, and a Z-score was computed for each individual voxel, as detailed in section *Regression analysis*.

We used the Computational Morphometry ToolKit[8] (CMTK) to align the various volumetric functional maps (gaze-tuned, self-oscillatory, visual response) onto a single reference brain-stack. The latter was obtained by averaging four different samples (Tg*(elavl3:*GCaMP6f), 6 dpf) imaged with the one-photon light-sheet microscope at high spatial resolution (voxel size 0.4x0.4x2 µm). We further used CMTK to compute the morphing transformation from our reference brain to the Tg(*elavl3*:GCaMP5G) stack of the Z-Brain atlas[9]. This allowed us to map the functional data onto

the Z-Brain Viewer and thus to position various clusters relative to labeled anatomical landmarks.

Phase and frequency analysis of the HBO oscillations was carried out using the Hilbert transform. This method allows one to extract the phase signal, associated with the dominant frequency, in a broadband time trace[10]. We implemented this mathematical tool on the differential HBO signal (left- minus right-circuit activity) to assign a phase $\varphi(t)$ to the HBO oscillatory dynamics. The angular frequency was then computed as the mean derivative of $\varphi(t)$; the HBO period distribution was obtained by performing the same operation over a sliding window of width 2π (Fig. 3g). To determine the phase-dependent response curve (Fig. 4k), the local phase $\varphi(t)$ was obtained through linear extrapolation of the Hilbert transform calculated over ~1 period, *i.e.* from t-20 s to t-1 s. This method guaranteed that the phase calculated at the instant of the flash was not affected by the flash-induced response itself. Phase-locking was assessed by evaluating the non-uniformity of the peri-stimulus phase distribution within a 10 s window centered on the stimulus, using the Rayleigh test. Given the large number of stimuli delivered in each run (n>50), we computed the p-value using Fisher's approximation[11]: $p = e^{-z}$, where z is the score given by Rayleigh test. Under this approximation, the critical Z-score beyond which the phase follows a non-uniform distribution with p<0.01 is 4.6 (Supplementary Fig. 8).

Optogenetics

Six dpf Tg(*elavl3*:GAL4; *UAS*:ChR2-mCherry) fish were immobilized in a drop of 2 % low melting point agarose deposited on a petri dish, and their eyes were freed. Larvae were imaged with a 10X objective on an upright microscope (Zeiss Examiner D1, Zeiss, Germany). Images were acquired at 10 Hz with a camera (ImageEM, Hamamatsu, Japan), allowing for subsequent extraction of the gaze sequence. We used a digital mirror device (DLP D4100, Vialux, Germany) to generate 2D illumination patterns for optogenetic stimulation. The DMD surface was relayed *via* a telescope onto the back-focal plane of the epifluorescent light-path of the microscope (Supplementary Fig. 6a). We used a high power white LED (Prizmatix, Israel) and a TIR prism

(Lida optical and electronic, China) to illuminate the DMD surface. The white light was passed through a filter set in the epifluorescence light-path to select the spectral band necessary for ChR2 activation (bandpass 495 nm). The DMD and the camera were controlled in LabView (National Instruments, Austin, TX, USA) and Matlab (Mathworks, Natick, MA, USA). The light source, camera recording and light-patterning were synchronized *via* pClamp (Digidata 1440A, Molecular Devices, Fremont, CA, USA).

The stimulation protocol was the following: pairs of bilaterally symmetric 47x57 μm^2 regions were illuminated alternately for 2.75 s with a period of 18 s. This alternation was repeated 5 times before moving to a new regions pair. The order of the 20 targeted pairs was randomized before each trial. As previously reported[4], activation of the rh4-6 region of the hindbrain evoked conjugate ipsiversive saccades. In some animals however, activation of the hindbrain failed to evoke any significant oculomotor response. This reflects the random variegation in the Tg(*elavl3*:GAL4; *UAS*:ChR2-mCherry) line, which results in diminished hindbrain ChR2 expression in a fraction of the animals. These non-responsive animals (Z-score<1 in rh4-6 regions) were excluded from further analysis, yielding a total of 8 (out of 19) larvae from which the analysis was performed.

To estimate the photostimulation volume, we implemented the same illumination protocol using a Tg(HuC:gal4; UAS:Kaede) line, in which the photoconvertible protein Kaede is expressed near pan-neuronally. This approach was previously shown to provide a close estimate of the illuminated volume[12]. Seven regions, selected at different locations on the checkerboard, were illuminated for 40s using a mercury arc lamp as the light source for the DMD using the whole spectrum <515nm of the mercury arc lamp. After photoconversion, whole brain stacks of red and green Kaede fluorescence were acquired using a spinning-disk confocal microscope (Intelligent Imaging Innovations, Denver, CO, USA) and the ratio of red/green fluorescence was calculated for further analysis (see Supplementary Fig. 6c).

In order to position optogenetically-targeted regions with respect to the formerly identified gaze-tuned circuit (Supplementary Fig. 6b), we successively imaged a unique reference fish (Tg(*elavl3*:GCaMP6), 6 dpf), successively with one-photon light-sheet (3D stack) and bright-field (2D image, z=160 μm from the dorsal skin) illumination at similar 10X magnification. We performed a 2D registration of the 19 tested fish onto the bright field reference image using the Matlab built-in functions *imregtform*, *fitgeotrans* and *imwarp*. The gaze-tuned 3D functional map (shown in Figure 2) was further registered onto the reference brain-stack using *CMTK*. For each fish, we computed the optogenetically-induced gaze shift $\Delta\theta_{gaze}(t_{stim}) = \theta_{gaze}(t_{stim} + 1) - \theta_{gaze}(t_{stim} - 1)$ for all stimulation onset times t_{stim}. For each targeted pair of regions, we computed a Z-score $= < \Delta\theta_{gaze}(t_{stim}) >_{all\ stim} / std(\Delta\theta_{gaze})$, where the standard deviation was calculated over the entire run. Averaging was done over all ten stimulations with ipsiversive gaze shifts counted positively and contraversive gaze shifts counted negatively.

To estimate the illumination volume, we aligned the 35 photo-converted volumes extracted from 5 different ratiometric stacks (7 regions were illuminated in each sample). The mean volume, shown in Supplementary Fig. 6d, appears to faithfully reflect the 2D rectangular shape imposed by the DMD, while being essentially invariant along the z-axis. This observation, which is consistent with the low NA of the illumination system, guarantees that the activation efficiency should be relatively insensitive to the axial location of the neurons within the brain. .

<u>Neuronal model</u>

Rate model equations of the HBO dynamics:

The mean firing rate of the left/right HBO subcircuits were described by 4 differential equations:

$$\tau \frac{dr_{L/R}}{dt} = -r_{L/R} + \Phi[W_E r_{L/R} - W_I r_{R/L} - \Gamma a_{L/R} + I_0 + I_{L/R}(t)] + \xi_{L/R}(t) \qquad (2)$$

with

$$\tau_A \frac{da_{L/R}}{dt} = -a_{L/R} + r_{L/R} \qquad (3)$$

where $W_E r_{L/R}$ and $W_I r_{R/L}$ denote the recurrent excitation and reciprocal inhibition, the terms $-\Gamma a_{L/R}$ account for adaptation current, I_0 and $\xi_{L/R}$ are constant and white noise (standard deviation σ) inputs originating from non-visual projections, $I_{L/R}(t)$ are visually-evoked currents. The function Φ was taken as the simplest form consistent with the non-negative spiking constraint: $\Phi(x > 0) = x$ and $\Phi(x < 0) = 0$ otherwise. Equations (2) and (3) were numerically integrated using Euler's method. In such a dynamic system, the self-excitation and adaptation currents produce the rise and fall of activity in one population, while inhibition ensures that only one population can be active at a time. Within a finite range of parameters, the system exhibited self-sustained anti-phasic oscillations in the absence of visual inputs ($I_{L/R}$= 0) . We further adjusted the values of the relaxation times such that the endogenous oscillatory period matched the mean saccadic period (T_{endo}=25 s). The corresponding parameters read:

$W_E = 3.5\ A.s$; $W_I = 0.5\ A.s$; $\Gamma = 2.9$; $\tau = 2\ s$; $\tau_A = 1\ s$; $I_0 = 20\ A$;

The current noise amplitude σ directly controlled the temporal regularity of the oscillations, which could be characterized by the phase-noise amplitude σ_f (Supplementary Fig. 9). This quantity, defined as the standard deviation of the distribution of phase increment over a 1s interval, was measured at σ_f=0.55$\pm$0.11 in experiments (fixed-eyes conditions, no visual stimulation, N=8 fish). We set the phase-noise at a slightly smaller value (σ_f ~ 0.2-0.4) in order to quantitatively reproduce all synchronization processes.

A unilateral step-increase in illumination was assumed to elicit a 0.3 s-long positive pulse of current followed by a constant input current on the ipsilateral HBO circuit. The intensity A_t of the pulse was proportional to the illumination increment, and the plateau intensity A_c was proportional to the stimulus intensity. A unilateral step-decrease in illumination further elicited a similar 0.3 s-long positive pulse of current on the contralateral HBO circuit. Data shown for flash stimuli were obtained with A_t=100 A. For alternated stimulation, we used A_t= 100 A and A_c=400 A.

To evaluate amplitude- and phase-response curves, we successively produced free-running and flash-evoked traces using identical noise signals (Supplementary Fig. 10). The flash-induced

response was then characterized by the difference in activity and the phase-offset between both configurations measured 1.5 s after the stimulus. This operation was repeated for different noise realizations and stimulation times.

We modeled the effective visual stimulus received from a distant light source by each eye as a slowly varying function of its azimuth θ_s relative to the fish caudo-rostral axis. The perceived stimulus was maximum when the source was aligned with the eye's optical axis, *i.e.* for θ_s=+/-80°, and was null beyond the monocular visual field[13] (total angular range 163°). The value of the left and right visually evoked current as a function of the light source azimuth thus reads:

$$I_L(\theta_s) = 100 * cos((\theta_s - 80°)\frac{180°}{163°})\Theta(\frac{163°}{2} - |\theta_s - 80°|)$$

$$I_R(\theta_s) = 100 * cos((\theta_s + 80°)\frac{180°}{163°})\Theta(\frac{163°}{2} - |\theta_s + 80°|) \qquad (4)$$

where Θ is the Heaviside step function.

In this second configuration, we assumed that the light source produced an identical stimulation on both eyes (no stereo-visual cue), and that the associated input current was a slowly decaying function of the angle between the gaze orientation θ_{gaze} and the source azimuth θ_s:

$$I_L(\theta_{gaze}, \theta_s) = I_R(\theta_{gaze}, \theta_s) = exp(-\left((\theta_{gaze} - \theta_s)/\sigma_{source}\right)^2/2)$$

with $\sigma_{source} = 45°$. Saccade-induced gaze oscillations were accounted for by switching the gaze orientation between θ_{gaze}=-15° (when $r_L > r_R$, *i.e.* leftward gaze) and θ_{gaze}=+15° (when $r_R > r_L$, i.e. rightward gaze), which in turn elicited a rhythmic modulation of the received currents (Supplementary Fig. 11). In order to account for background adaptation, the input currents at both angular positions were offset by the weaker one.

Supplementary Figure 1 | Behavioural assays: ocular saccades and eyes/tail coordination.

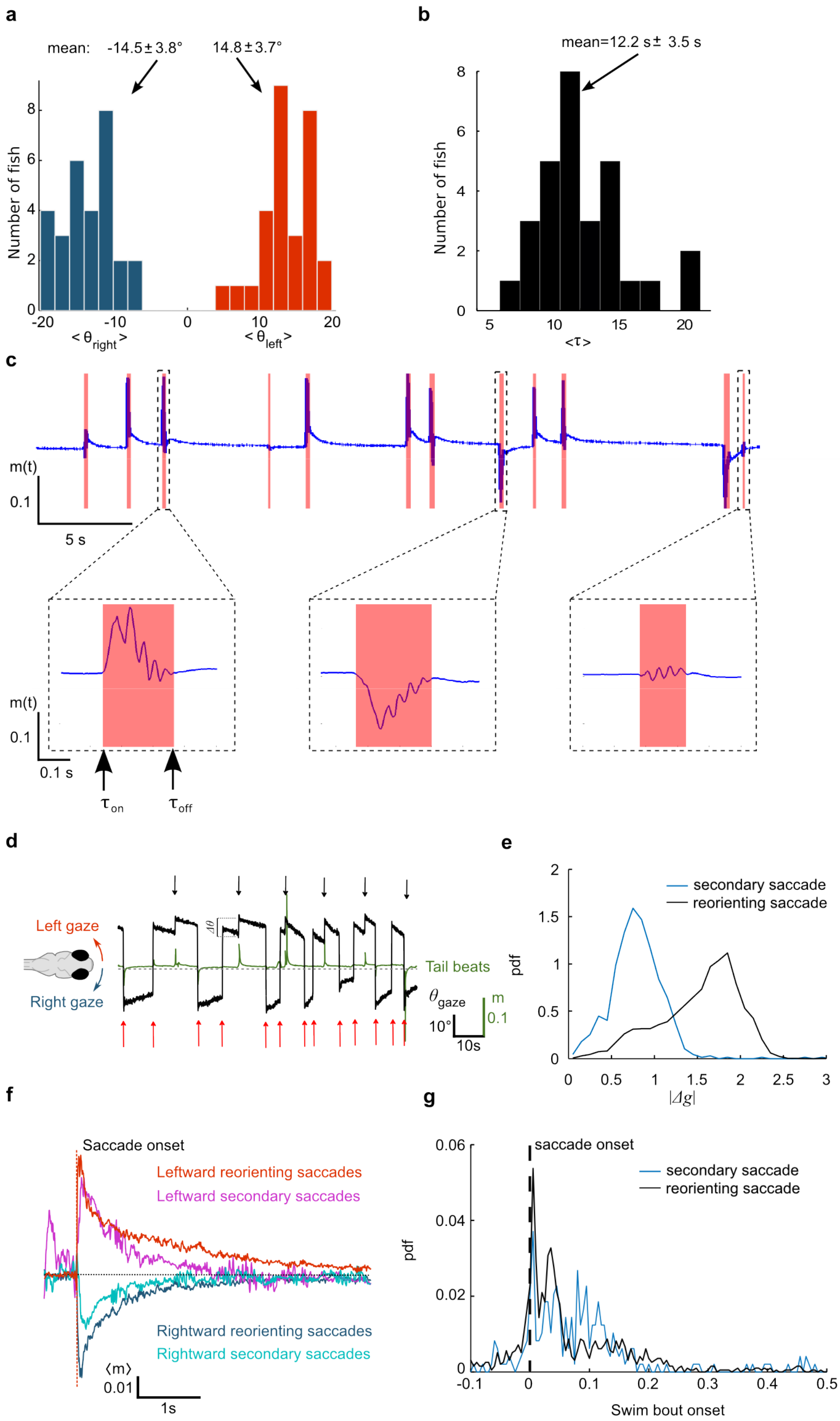

Supplementary Figure 1 | Behavioural assays: ocular saccades and eyes/tail coordination. **a**, Histograms of the mean gaze angles measured immediately after rightward ($< \theta_{gaze\ right} >$) and leftward ($< \theta_{gaze\ left} >$) saccades (N = 29 fish). These angles are used to compute the normalized gaze angle g (see Supplementary Methods). **b**, Histogram of the mean gaze fixation times (N = 29 fish). **c**, Example tail-deflection signals (normalized area moment m(t), see Supplementary Methods). Each red region corresponds to a discrete swim-bout from which the onset and offset times (τ_{on} and τ_{off}) are extracted, as well as a turning score *M* defined as the integral of *m(t)* between τ_{on} and τ_{off}. **d-g,** Comparison between reorienting and secondary saccades. **d**, Example traces of the gaze angle and tail deflection signals. The arrows points to reorienting (red) or secondary (black) saccades. **e**, PDF of the absolute gaze shift amplitude $|\Delta g|$ associated with reorienting (black) and a secondary (blue) saccades. **f**, Mean peri-saccadic tail deflection signal averaged over leftward reorienting (red) or secondary (magenta) saccades, and rightward reorienting (blue) or secondary (pale blue) saccades. **g**, Peri-saccade PSF of swim-bouts onset times ($\tau_{on} - t_{sac}$) for reorienting saccades (black) and secondary (blue) saccades. The saccade generally precedes the tail beat : 88% of the tail beats that occur within 0.3s of a saccade are triggered after the saccade onset.

Supplementary Figure 2 | Behavioural assays: light-induced bias in gaze-dynamics.

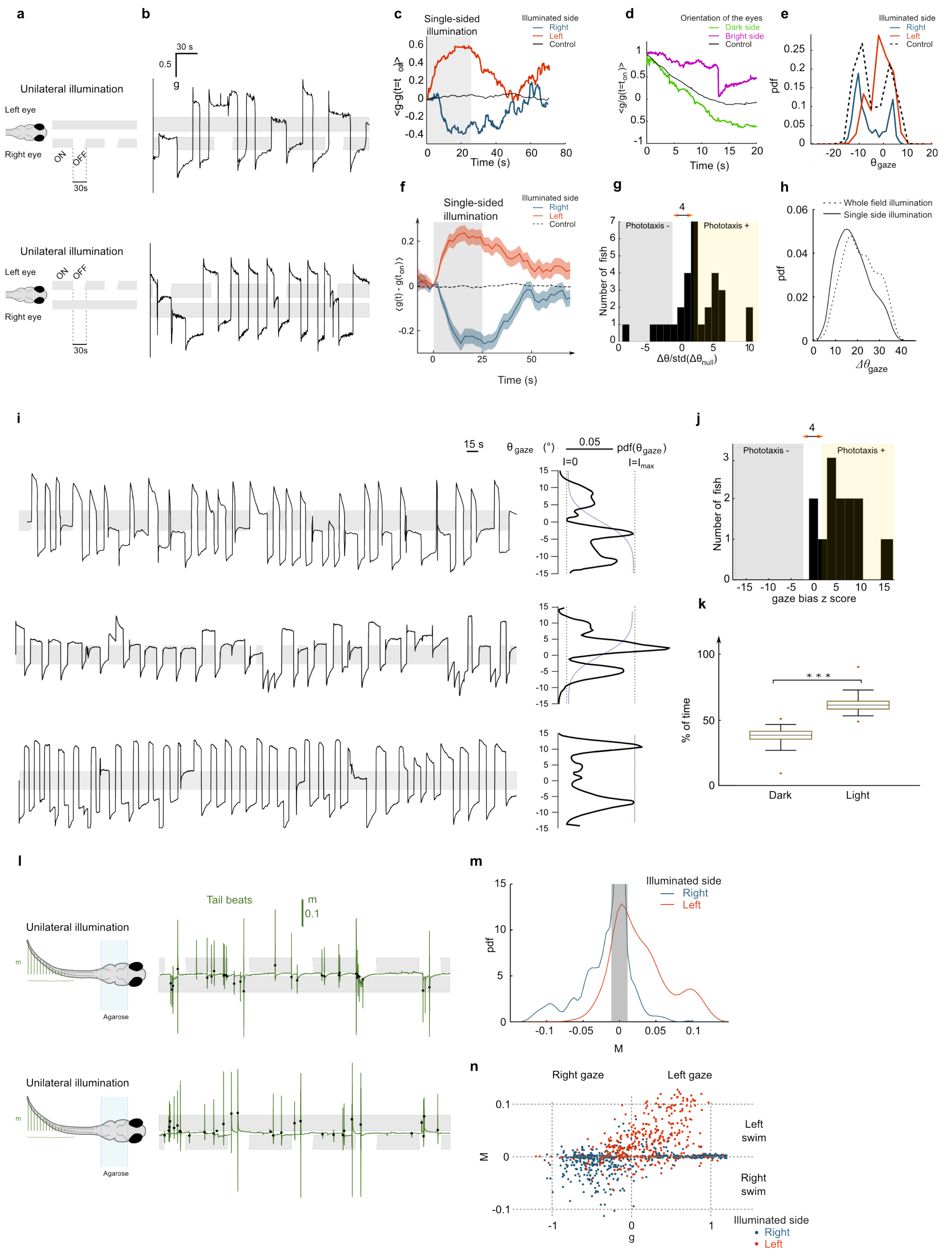

Supplementary Figure 2 | Behavioural assays: light-induced bias in gaze-dynamics. a, Scheme of the unilateral extinction experiment. **b**, Gaze signals during a series of unilateral light extinctions. The greyed areas indicate the stimulus sequence delivered to each eye. **c**, Corresponding trial-averaged normalized gaze signal $< g(t) - g(t = t_{on}) >_{all\,stim}$following of the light on the right (red) and left (blue) eye. The black line is the control, i.e. the mean gaze signal following 100 randomly distributed times t_{on}. **d**, Trial-averaged gaze decay $< g(t)/g(t = t_{on}) >_{all\,stim}$ following unilateral extinction. In magenta, averaging was performed over trials for which the gaze is orientated towards the bright side at $t=t_{on}$. In green, the gaze is oriented towards the dark side at $t=t_{on}$. The black curve is the control (averaged over randomly distributed onset times). These data indicate that unilateral extinction both lengthens gaze fixation towards the bright side (slower decay) and shortens gaze fixation (faster decay) toward the dark side. **e**, PDF of the gaze during periods of unilateral visual stimulation for the same animal. In red (resp. blue), the light is ON on the left (resp. right) eye. The black dashed curve is the gaze distribution during bilaterally symmetric illumination. **f**, Trial-averaged gaze shift signal $< g(t) - g(t = t_{on}) >_{all\,stim}$ following unilateral extinction on left (red) or right (blue) eye at $t = t_{on}$. The shaded region represents the s.e.m. **g**, Histogram of the mean gaze shift (normalized by the standard deviation of the control) during unilateral extinction phases (N=33 fish). Gaze shifts are counted positively (resp. negatively) when oriented towards (away) from the bright side. Grey and yellow shaded areas correspond to animals displaying statistically significant gaze bias away and towards the illuminated side, respectively (two-tailed t-test, Z-score>2, $p<0.05$). **h**, PDF of the saccade-induced gaze variation during periods of unilateral illumination and whole field illumination (dotted line). **i**, Left : Example gaze signals for a virtual rightward (top), leftward (middle), uniform (bottom) illumination. Grey areas indicate bright illumination periods (intensity larger than half its maximal value). Right : corresponding gaze-angle PDFs. The blue curves show the imposed virtual illumination profile. **j**, Histogram of the gaze-biased Z-score (see Supplementary Methods). As in (g), the grey and yellow shaded areas correspond to animals displaying statistically significant gaze bias away and towards the illuminated side, respectively

(two-tailed t-test, Z-score>2, p<0.05). **k**, Time fractions spent with the gaze oriented towards the dark and bright directions (N=13 fish). *** p<0.001, paired-sample t-test. **l**, Tail-deflection signals (m) during a series of unilateral light extinctions. Greyed areas indicate the stimulus sequence delivered to each eye. **m**, PDF of the tail-beat turning index M during periods of unilateral visual stimulation for animals displaying light-induced gaze-bias (N=5). In red (resp. blue), the light is ON on the left (resp. right) eye. **n**, Individual tail-beats turning score M *vs* normalized gaze angle *g* during periods of unilateral visual stimulation.

Supplementary Figure 3 | Long-lead burst neurons, HBO activity during secondary saccades and putative circuit architecture.

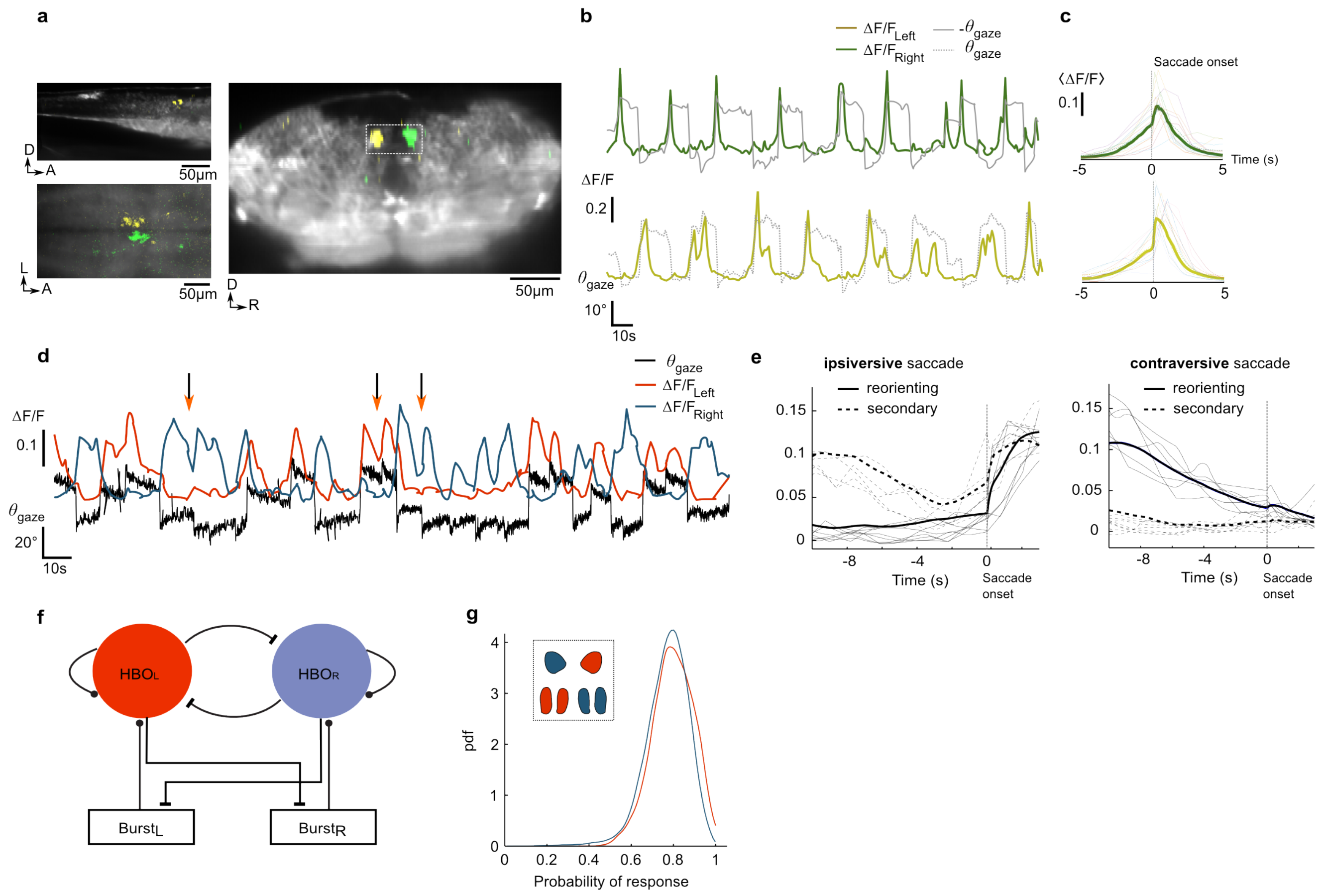

Supplementary Figure 3 | Long-lead burst neurons, HBO activity during secondary saccades and putative circuit architecture. a, Sections of the velocity-tuned map (averaged over seven fish) showing the long-lead burst neurons located in two well-defined clusters in the dorsal region of rh 7. **b**, Example traces of these two clusters and associated gaze signals. **c,** Mean peri-saccadic signals. A systematic increase of activity is observed ~2-3 s before the ipsiversive saccade onset. **d-e**, Secondary saccades**. d**, Example traces of the left- and right-HBO activity and the gaze signal. When two successive saccades occur in the same direction (arrows), the active circuit displays a transient rebound. **e**, Mean peri-saccade signals of the HBO subnetworks for reorienting saccades (solid line) and secondary saccades (dashed line). The data are shown separately for ipsiversive (left) and contraversive (right) saccades. **f**, Scheme of a putative network architecture accounting for the HBO self-oscillatory dynamics and its interaction with the saccade generator circuit (burst cells). Each HBO subcircuit send inhibitory projections to the contralateral burst cells. It receives afferent excitatory inputs from ipsilateral burst neurons. **g**, Robustness of the HBO gaze-tuned characteristics. For each voxel of the right (blue) and left (red) HBO, we computed the fraction of ipsiversive saccades for which a significant post-saccadic increase in $\Delta F/F$ (larger than one standard deviation) is observed. The plot shows the PDF of this quantity for all the HBO voxels.

Supplementary Figure 4 | Delineating the self-oscillatory circuit in eyes-fixed experiments.

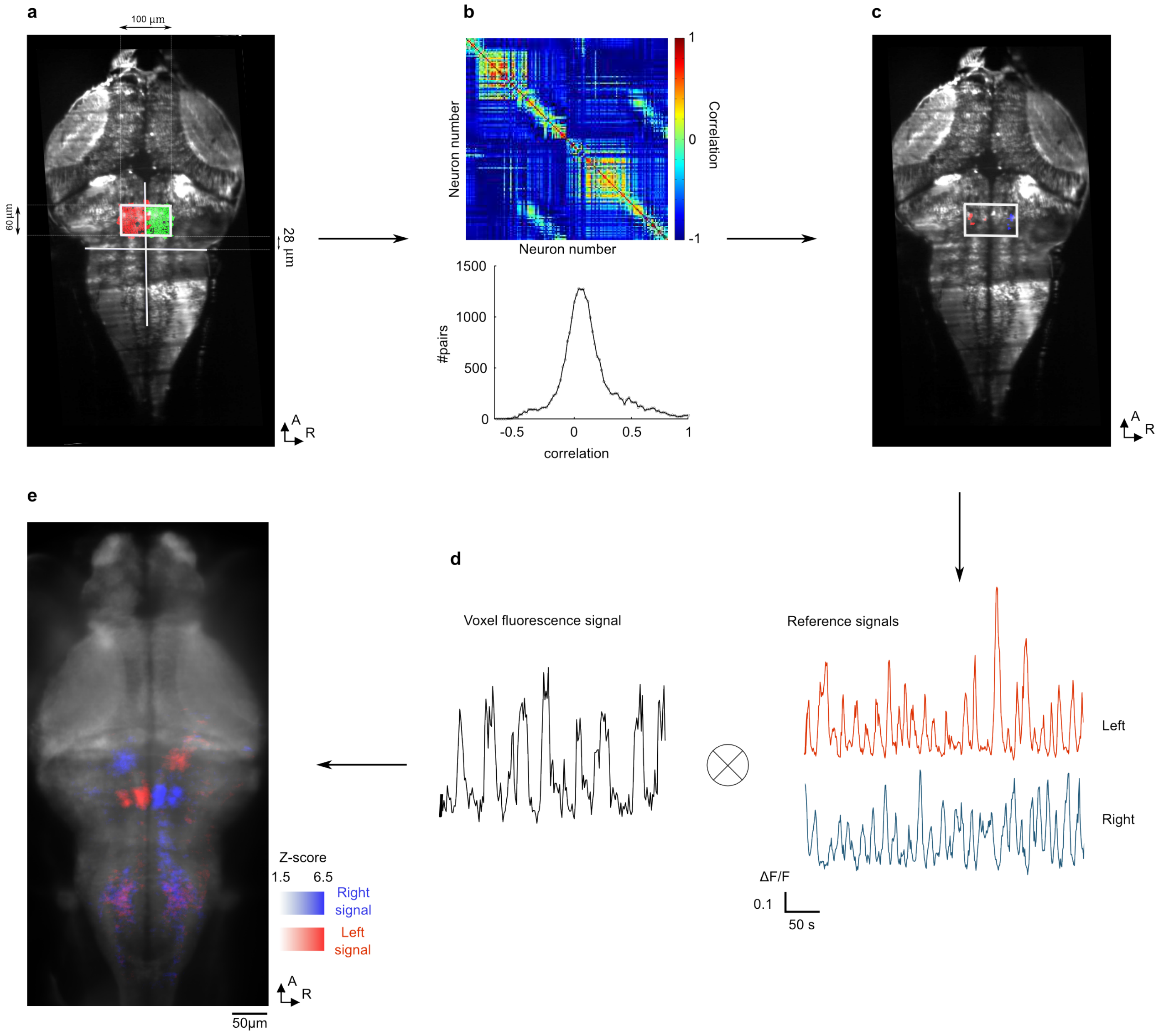

Supplementary Figure 4 | Delineating the self-oscillatory circuit in eyes-fixed experiments. **a**, Pre-selected volume encompassing the gaze-tuned rostral hindbrain circuit. The rostral border of the 100x60x27µm volume is positioned 28 µm from the hindbrain constriction axis. **b**, Pearson correlation matrix (top) and correlation histogram (bottom) of the neuron pairs within the pre-selected volume. **c**, Neurons engaged in the 10 most highly anti-correlated pairs. **d**, Reference signals are computed as the the mean ΔF/F of the left and right previously selected neurons, and then used as regressors to map the self-oscillatory network across the entire volume. **e,** Z-projection of the resulting self-oscillatory hindbrain network (N=8 fish). Color encodes tuning (Z-score) to the left (red) and right (blue) reference signals.

Supplementary Figure 5 | Gaze-tuned maps in the absence of visual stimulation and comparison between gaze-tuned, visually responsive and self-oscillatory maps.

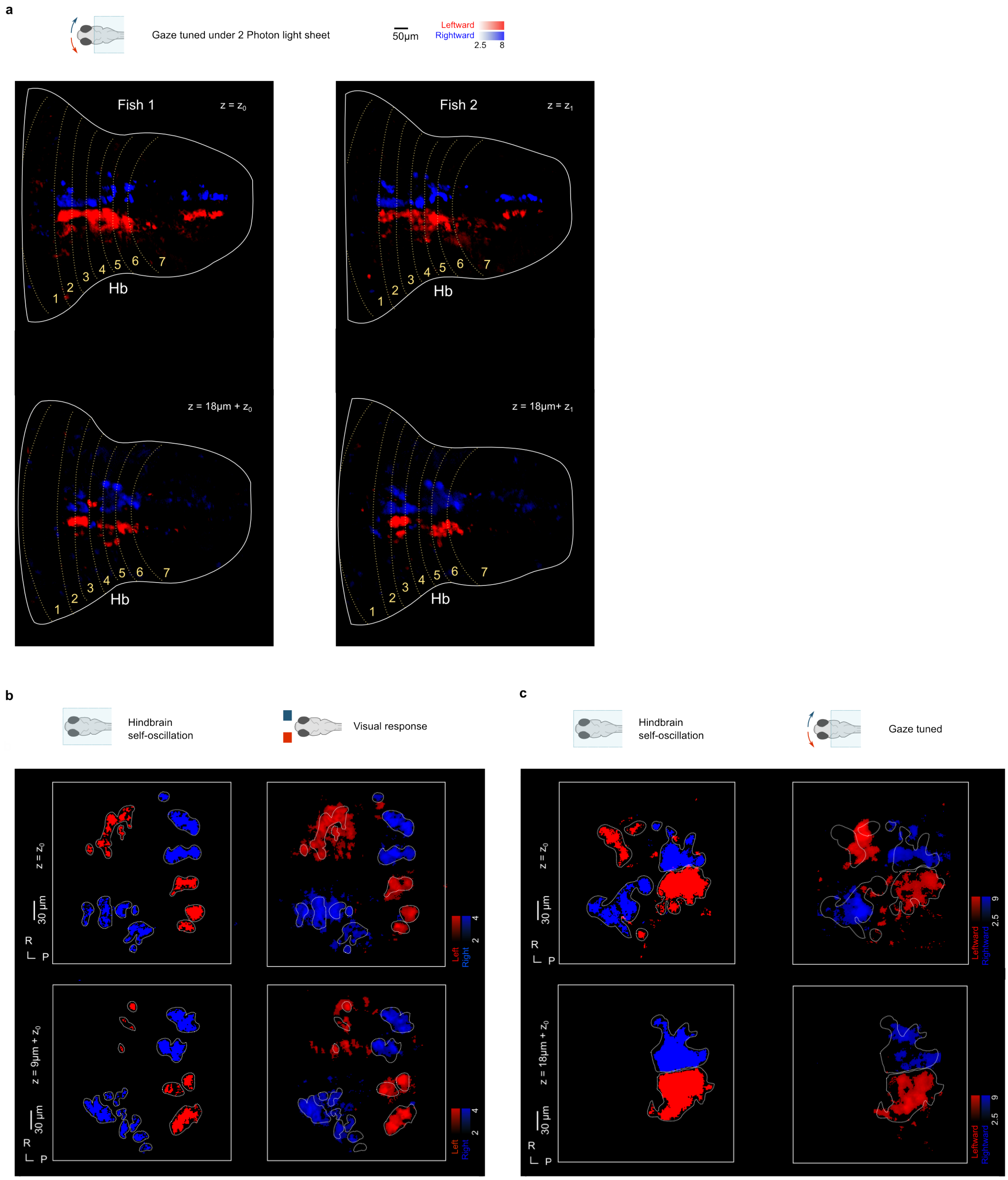

Supplementary Figure 5 | Gaze-tuned maps in the absence of visual stimulation and comparison between gaze-tuned, visually responsive and self-oscillatory maps. a, Two dorso ventral sections of gaze-tuned maps obtain on two different fish under 2-photon light-sheet imaging. Experiments were performed in the absence of visual stimulation. **b**, Comparison on the same fish, for two different brain sections, of the self-oscillatory hindbrain population (left) and the visually responsive neurons (right). The white contour line delineates the self-oscillatory region, *i.e.* the voxels that display a Z-score>3.5 when correlating with the reference oscillatory signal (see Supplementary Methods). **c**, Comparison on the same fish, for two different brain sections, of the self-oscillatory hindbrain population (left) and the gaze-tuned neurons (right). The white contour line is defined as in b. These data were obtained by performing experiments with a larva whose eyes were successively tethered then free.

Supplementary Figure 6 | Optogenetic assay.

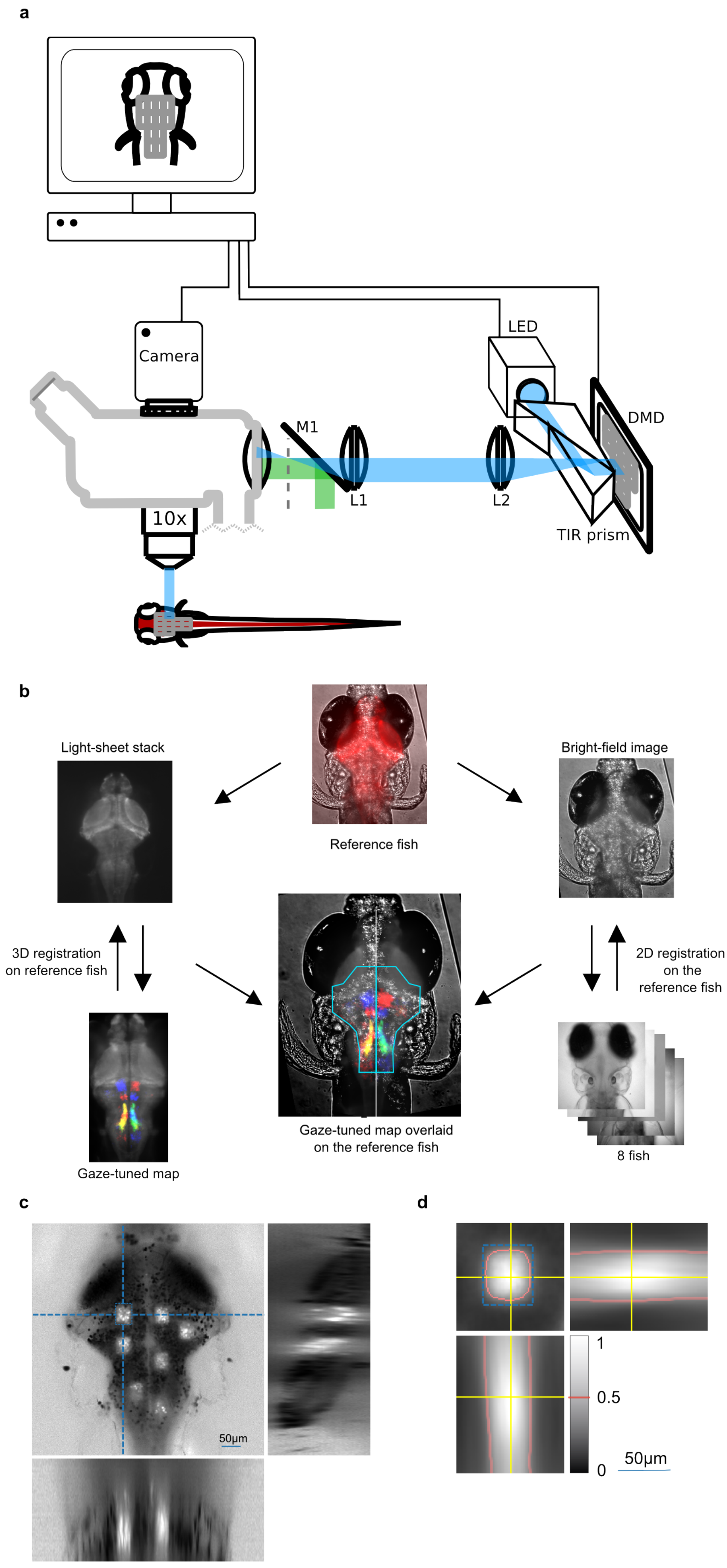

Supplementary Figure 6 | Optogenetic assay. a, Schematic layout of the optogenetic experiment. A checkerboard pattern is drawn over the brain image and the rectangles are sequentially displayed on a digital mirror device (DMD). The pattern on the DMD is imaged *via* the lenses L1 (f=40 mm) and L2 (f=80 mm) onto a confocal plane (dashed line) of the epifluorescent light path and ultimately onto the targeted region of the fish brain. A total internal reflection prism (TIR) is used to illuminate the DMD with the stimulation light source (LED). The beam splitting mirror M1 (30% reflection, 70% transmission) allows one to simultaneously use a halogen light source for epifluorescence illumination and simultaneous eyes monitoring. Camera recording and optogenetic activation are synchronized by the computer. **b,** Schematic description of the morphological registration method used to merge different optogenetic experiments and to overlay the activated regions onto the gaze-tuned functional map. **c,** Sections of one photoconverted volume using a Tg(HuC:gal4; UAS:Kaede) larva. Pixel intensity represents the red/green flurescence ratio. The larva was exposed to UV illumination using the same illumination pattern as in the optogenetic assay, but for 7 regions only. **d**, Sections of the average illumination volume, obtained by registrating 5 larvae, *i.e.* 35 volumes in total. The pixel intensity denotes the red/green fluorescence ratio normalized between 0 (outside the volume) and 1 (at the center). The isovalue line corresponding to 0.5 is shown in red. In c and d, the dotted blue rectangle indicates the targeted area.

Supplementary Figure 7 | Visual stimulation on one eye and HBO intensity tuning curve.

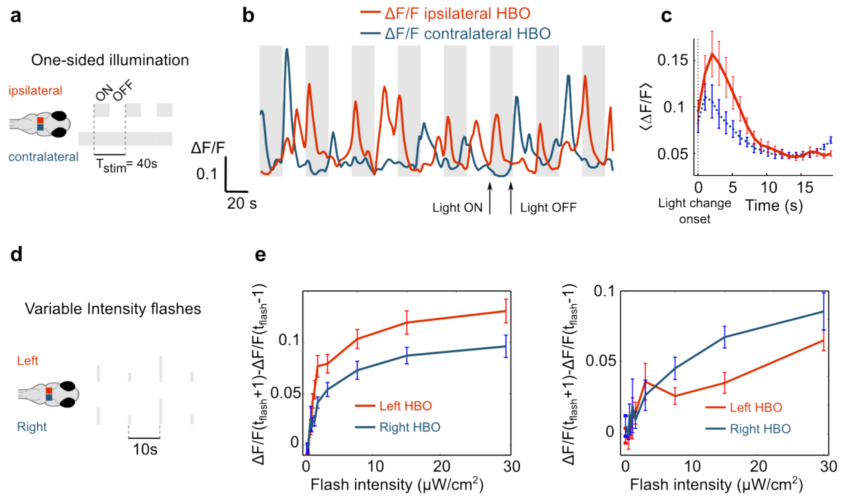

Supplementary Figure 7 | Visual stimulation on one eye and intensity tuning curve. a, One- sided stimulation experimental scheme. The illumination is maintained constant on one eye while it alternates between dark and bright every 20 s on the other eye. **b**, Response of the HBO populations to unilateral visual stimulation. The red (resp. blue) curve correspond to the ipsilateral (resp. contralateral) HBO with respect the eye exposed to the alternating stimuli. Greyed regions indicate the unilateral stimulation sequence. **c**, Red curve: trial-averaged response of the ipsilateral HBO to light-ON transitions. Blue curve: trial-averaged response of the contralateral HBO to light-OFF transitions. Data are averaged over N=8 fish. The error bars represent the s.e.m. These response curves suggest that the HBO receive both ipsilateral projections from the ON visual pathway and contralateral projections from the OFF visual pathway. **d**, The fish is submitted to a series of 350 flashes of varying intensities with a 10 s inter-flash period. **e**, Mean intensity response defined as $< \Delta F/F(t = t_{flash} + 1) - \Delta F/F(t = t_{flash} - 1) >$ as a function of the flash intensity. The error bars represent the s.e.m. The tuning curve is computed for each subcircuit and is shown for two fish.

Supplementary Figure 8 | Phase-locking and frequency entrainment of the HBO by periodic visual stimuli.

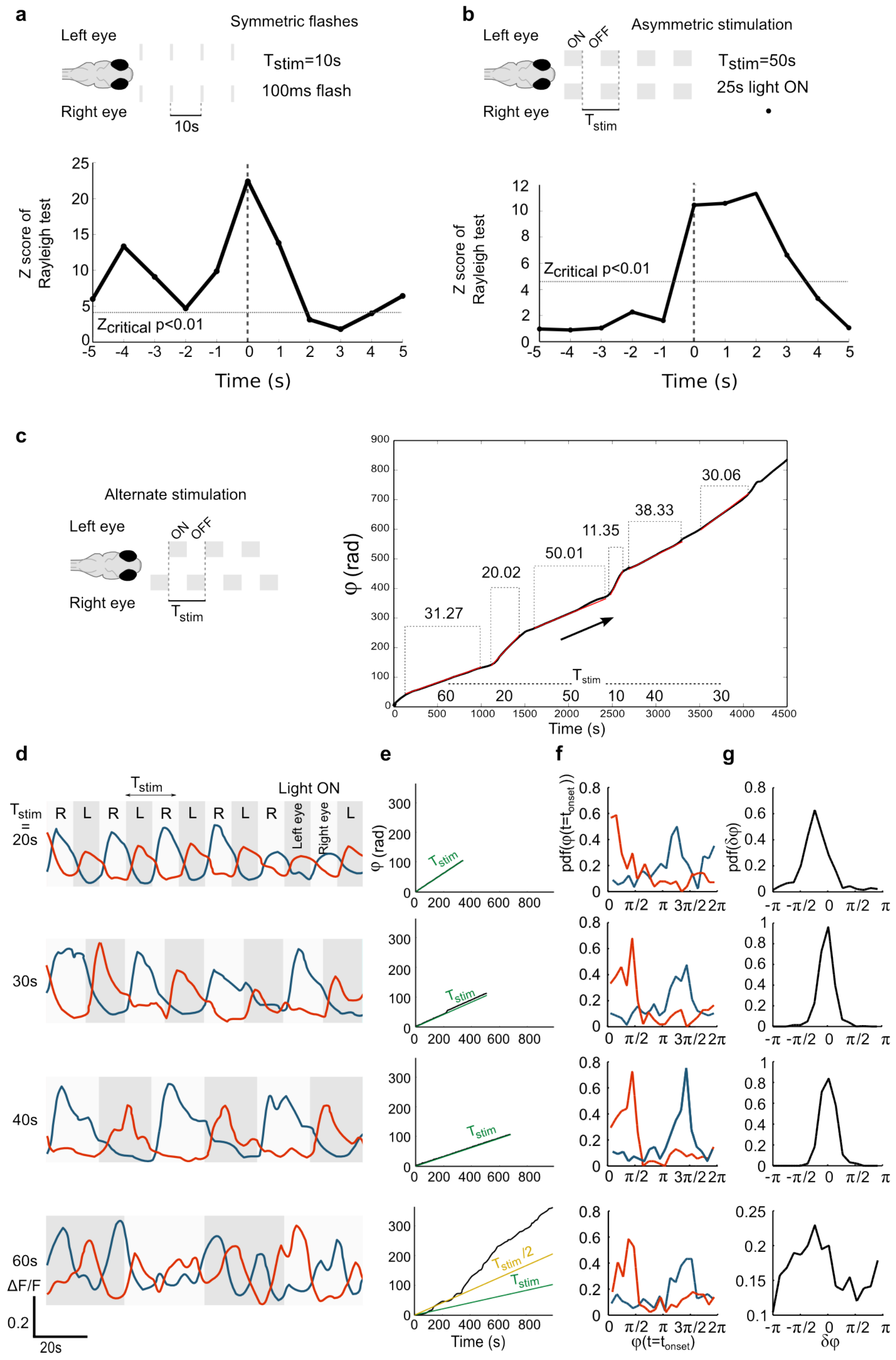

Supplementary Figure 8 | Phase-locking and frequency entrainment of the HBO by visual stimuli. **a**, Bilateral 100 ms-flash are delivered every 10 s. The curve shows the Z-score, computed from Rayleigh statistics, of the oscillatory phase distribution at each time point in a 10 s window centered on the stimulus (n=500 flashes, N=12 fish). The dashed line shows the critical Z-score ($p<0.01$) indicating threshold for significant phase concentration. This data indicates a strong visually induced phase concentration of the HBO oscillation. **b**, Same curve as (a) for bilateral light-ON/light-OFF stimulation (n=146 flashes, N=5 fish). Illumination onset induces a transient phase concentration. **c**, Evolution of the HBO phase signal during one run, upon antisymmetric alternated stimulation at different frequencies. Changes in the stimulation period (indicated above the x-axis) induce a change in the oscillatory dynamics (mean oscillatory periods are indicated above the curve), revealing frequency entrainment. **d**, Example signals of the two subpopulations for 4 stimulation frequencies. **e**, Evolution of the HBO phase (black curve). The phase of the stimulation $2\pi\ t/T_{stim}$ is shown in green. **f**, PDF of the HBO phase at light-ON (red) and light-OFF (blue) transitions. **g**, PDF of the HBO/stimulation phase-offset.

Supplementary Figure 9 | Effect of the current noise on the self-oscillatory signal in the neural model.

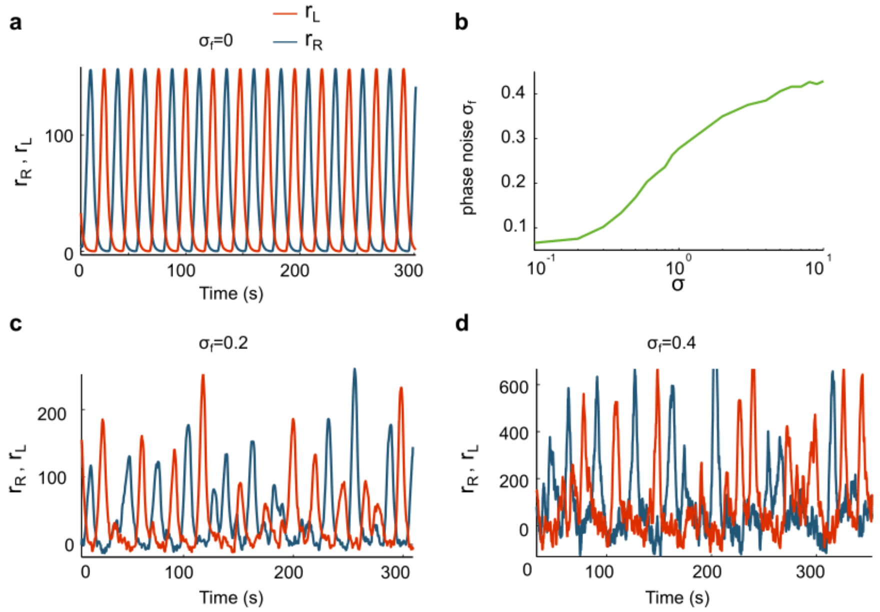

Supplementary Figure 9 | Effect of the current noise on the self-oscillatory signal in the neural model. a, Activity of the left and right simulated networks in the absence of current noise ($\sigma = 0$). **b**, Phase noise σ_f as a function of the current noise σ. The phase noise is defined as the standard deviation of the distribution of phase increments measured over a 1 s interval. **c-d,** Self-oscillatory signals for a phase noise σ_f= 0.2 and 0.4, *i.e.* for σ_f= 0.55 and 5, respectively.

Supplementary Figure 10 | Phase dependent responses and phase-locking in the network model.

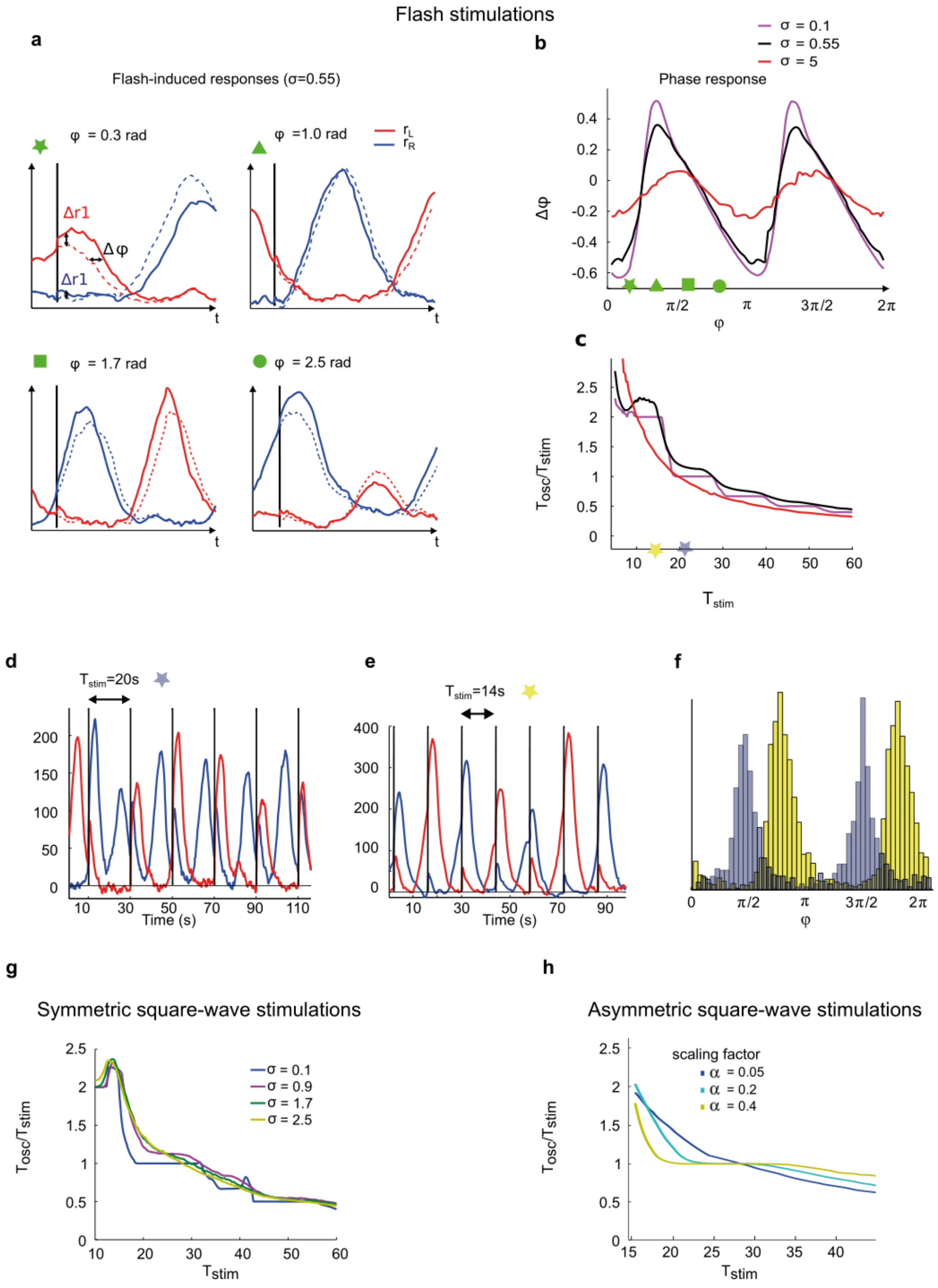

Supplementary Figure 10 | Phase-dependent visual responses and phase-locking in the network model. a, Example HBO responses to flashes delivered at 4 different oscillatory phases (neuronal model). The dotted curve shows the HBO trace when no flash is delivered. **b**, Phase response curves (PRC) for different noise levels. The green symbols indicate the phases at flash delivery in (a). **c**, Ratio of the oscillatory and stimulation periods, as a function of the stimulation period, for different noise values. The “Devil’s Staircase” shape of the curve is a signature of the HBO synchronization at integer multiples or rational fractions of the stimulus period. **d-e**, Oscillatory HBO signals upon periodic flash stimulation for (d) T_{stim} = 20 s and (e) T_{stim} = 14 s. In the first case, the stimulus period is slightly below the endogenous period (T_{endo} = 25s) and the HBO is entrained at the stimulus frequency. In the second case, T_{stim} is slightly larger than $T_{endo}/2$ and the HBO oscillates at half the stimulus frequency (see corresponding symbols in (c)). **f**, Phase histograms at flash times for these two configurations. When T_{stim}=20 s (grey), the two peaks correspond to negative values in the phase-response curve ($\Delta\varphi$>0), indicating that the stimuli constantly speed up the HBO oscillation, allowing the one-to-one synchronization process to occur. When T_{stim}=14 s, the phase distribution at flash-times is peaked at phase values such that $\Delta\varphi$<0 (panel **e**, yellow histogram). The stimuli slow down the HBO oscillation, leading to the period-doubling synchronization. **g**, Entrainment curve for symmetric square-wave stimulation at different noise values. Compared to the flash stimuli, the addition of a continuous component during one-half of the cycle extends the entrainment plateau for which T_{osc}~T_{stim}. **h**, Entrainment curve for asymmetric periodic stimulation. The different curves are obtained for different intensities of the stimulus, A_t=$\alpha*400$A and A_c=$\alpha*100$A (Supplementary Methods). In the symmetric case, $\alpha = 1$.

Supplementary Figure 11 | Phototactic behaviours in the rate model.

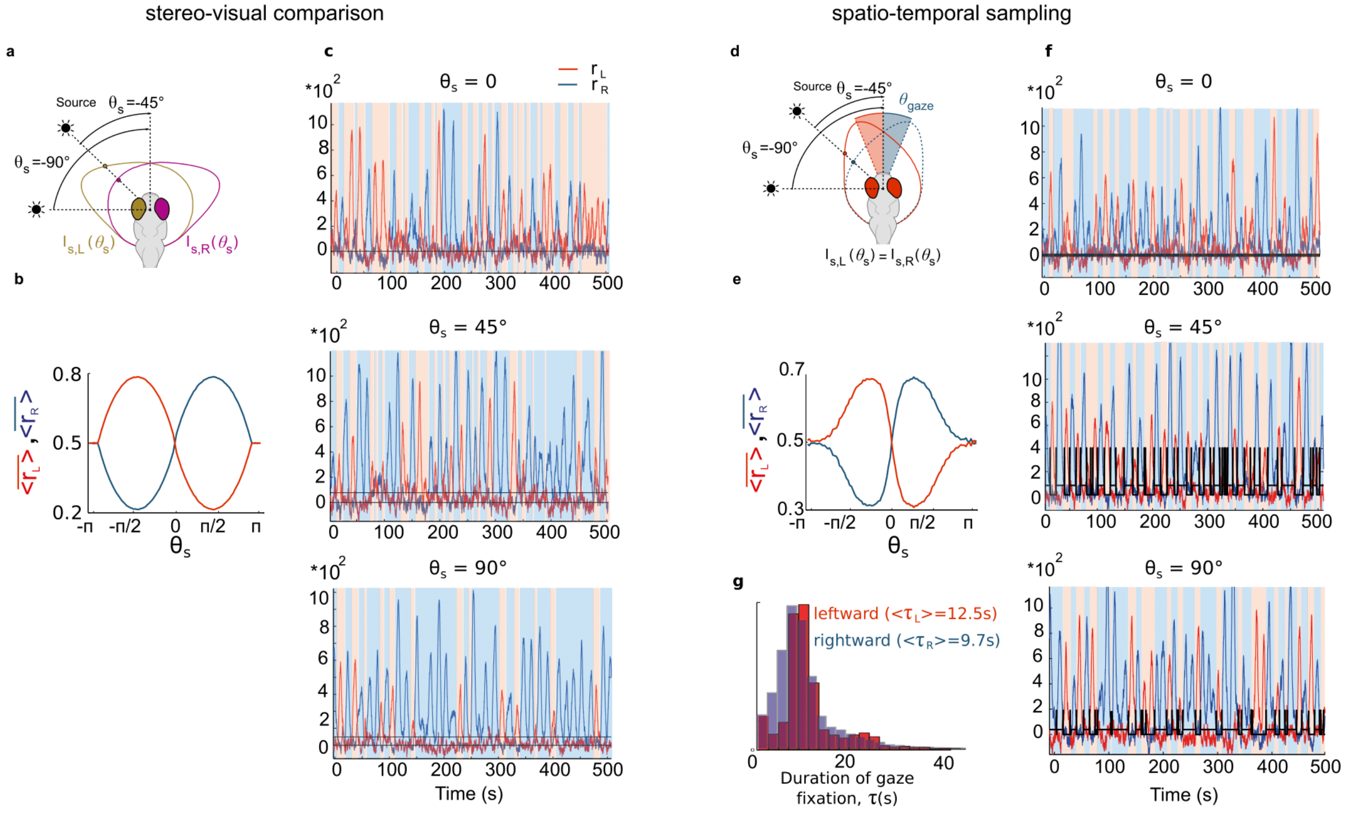

Supplementary Figure 11 | Phototactic behaviours in the rate model. a, Stereo-visual comparison (see Figure 6). **b**, Normalized firing rates of the left and right HBO circuits ($\overline{< r_{L/R} >} = \frac{<r_{L/R}>}{<r_L>+<r_R>}$) as a function of the light source azimuth θ_s. The HBO mean activity is biased ipsilaterally to the light source. **c**, Example traces of the simulated left and right HBO activity for three light-source azimuthal angles. The orange (blue) shaded areas delineate the periods during which $r_L > r_R$ ($r_R > r_L$). The black curves indicate the constant currents delivered to the two subpopulations. **d**, Spatio-temporal sampling (see Figure 6). **e-f**, Same as a-b. Each left-to-right or right-to-left transition induces a change in the current delivered symmetrically to the two subpopulations (black line in (f)). **g**, Histogram of time durations during which $r_L > r_R$ ($r_R > r_L$) corresponding to leftward (rightward) gaze-fixation periods for a source located 45° to the left. When the gaze is oriented towards the light source, the mean fixation period is increased.

Supplementary References:

1. Chen, T.-W. et al. Ultrasensitive fluorescent proteins for imaging neuronal activity. *Nature*, **499**, 295–300 (2013).
2. Higashijima, S.-I. et al. Imaging neuronal activity during zebrafish behavior with a genetically encoded calcium indicator. *Journal of neurophysiology*, **90**, 3986–3997 (2003).
3. Kimura, Y., Satou, C. and Higashijima, S.-I. V2a and V2b neurons are generated by the final divisions of pair-producing progenitors in the zebrafish spinal cord. *Development*, **135**, 3001–3005 (2008).
4. Schoonheim, P. J., Arrenberg, A. B., Del Bene, F., and Baier, H. Optogenetic localization and genetic perturbation of saccade-generating neurons in zebrafish. *J Neuroscience*, **30**, 7111–7120, (2010).
5. Wolf, S. *et al.* Whole-brain functional imaging with two-photon light-sheet microscopy. *Nat Methods*, **12**, 379–380 (2015).
6. Panier, T., *et al.* Fast functional imaging of multiple brain regions in intact zebrafish larvae using Selective Plane Illumination Microscopy. *Front Neural Circuits*, **7**, 65 (2013).
7. Miri, A., Daie, K., Burdine, R. D., Aksay, E. and Tank, D. W. Regression-based identification of behavior-encoding neurons during large-scale optical imaging of neural activity at cellular resolution. *J Neurophysiol*, **105**, 964–980 (2011).
8. http://www.nitrc.org/projects/cmtk/
9. Randlett, O. *et al.* Whole-brain activity mapping onto a zebrafish brain atlas. *Nat Methods*, **12**, 1039-1046 (2015).
10. Quian Quiroga, R., Kraskov, A., Kreuz, T. and Grassberger, P. Performance of different synchronization measures in real data: a case study on electroencephalographic signals. *Phys Rev E*, **65**, 041903 (2002).
11. Fisher, N. I. Statistical analysis of circular data. *Cambridge University Press*, (1995).
12. Arrenberg, A. B., Del Bene, F., & Baier, H. Optical control of zebrafish behavior with halorhodopsin. *Proceedings of the National Academy of Sciences*, **106**, 17968-17973 (2009).
13. Easter Jr, S. and Nicola, G. N. The development of vision in the zebrafish. *Dev Biol*, **180**, 646–663 (1996).
